# Supplementary material for: Cumulative Effects of Particulate Matter Pollution and Meteorological Variables on the Risk of Influenza-Like Illness
Source: Viruses. 2021 Mar 26;13(4):556. doi: 10.3390/v13040556 (PMC8065612; doi:10.3390/v13040556)
Supplement: Supplementary file 1 [file viruses-13-00556-s001.zip › Tables and figures.pdf]

Table S1. Reported influenza-like illness cases in Bialystok and Bialystok County divided by year and by age group.

| Age \ Year | 2013 | 2014 | 2015 | 2016 | 2017 | 2018 | 2019 | 2013-2019 |
|---|---|---|---|---|---|---|---|---|
| **0-4** | 11,092 | 8,982 | 10,041 | 14,794 | 16,092 | 10,606 | 10,396 | **82,003** |
| **5-14** | 10,574 | 7,869 | 9,084 | 14,064 | 14,350 | 10,038 | 7,267 | **73,246** |
| **15-64** | 25,332 | 16,376 | 18,966 | 27,674 | 31,900 | 22,752 | 16,559 | **159,559** |
| **65+** | 4,716 | 2,563 | 3,224 | 4,975 | 6,226 | 5,711 | 3,764 | **31,179** |
| **Total** | **51,714** | **35,790** | **41,315** | **61,507** | **68,568** | **49,107** | **37,986** | **345,987** |

Table S2. Annual and 24-hour concentrations of particulate matter (μg/m$^3$) in Bialystok and the Bialystok County.

| | | | | 24-hour concentrations | | | | | | |
|---|---|---|---|---|---|---|---|---|---|---|
| **Year** | | Annual mean | SD | Min | P25 | Median | P75 | Max | n (%) > WHO daily guidelines | n (%) missing |
| **2013** | $PM_{2.5}$ | 22.9 | 11.1 | 3.8 | 15.2 | 20.3 | 28.1 | 75.2 | 122 (35%) | 21 (6%) |
| | $PM_{10}$ | 23.2 | 13.7 | 3.9 | 13.9 | 20.3 | 28.8 | 96.5 | 13 (4%) | 0 |
| **2014** | $PM_{2.5}$ | 24.9 | 11.7 | 5.9 | 16.0 | 22.9 | 32.8 | 69.7 | 151 (42%) | 4 (1%) |
| | $PM_{10}$ | 26.5 | 15.8 | 5.8 | 15.2 | 22.1 | 33.3 | 97.1 | 30 (8%) | 0 |
| **2015** | $PM_{2.5}$ | 24.9 | 14.7 | 6.1 | 15.0 | 19.9 | 31.6 | 91.0 | 125 (36%) | 16 (5%) |
| | $PM_{10}$ | 25.3 | 18.1 | 4.7 | 13.1 | 19.6 | 30.4 | 115.4 | 40 (11%) | 0 |
| **2016** | $PM_{2.5}$ | 19.8 | 9.1 | 3.3 | 12.9 | 18.1 | 25.2 | 65.2 | 77 (26%) | 65 (22%) |
| | $PM_{10}$ | 20.2 | 12.5 | 1.7 | 11.8 | 16.7 | 24.7 | 86.9 | 12 (3%) | 0 |
| **2017** | $PM_{2.5}$ | 21.0 | 12.9 | 2.7 | 12.9 | 17.9 | 25.8 | 129.7 | 92 (27%) | 23 (7%) |
| | $PM_{10}$ | 20.2 | 14.4 | 2.7 | 11.5 | 17.0 | 24.2 | 139.1 | 12 (3%) | 0 |
| **2018** | $PM_{2.5}$ | 24.0 | 13.3 | 2.4 | 14.2 | 21.4 | 29.9 | 88.4 | 132 (37%) | 9 (3%) |
| | $PM_{10}$ | 22.1 | 14.6 | 2.8 | 11.9 | 18.2 | 27.9 | 97.4 | 19 (5%) | 1 (<1%) |
| **2019** | $PM_{2.5}$ | 19.0 | 9.3 | 2.9 | 12.8 | 17.6 | 22.3 | 64.4 | 68 (19%) | 3 (1%) |
| | $PM_{10}$ | 18.6 | 10.1 | 2.7 | 11.8 | 16.8 | 22.5 | 91.2 | 3 (1%) | 0 |

Abbreviations: $PM_{2.5}$, particulate matter with aerodynamic diameters of 2.5 μm or less; $PM_{10}$, particulate matter with aerodynamic diameters of 10 μm or less; SD, standard deviation

Table S3. Spearman's correlation coefficients between the environmental factors and the incidence of influenza-like illness.

| | Number of cases | Temperature (°C) | Relative humidity (%) | Absolute humidity (g/m³) | Wind speed (m/s) | Precipitation (mm) | Precipitation duration (hours) | Sunshine duration (hours) | $PM_{2.5}$ (μg/m³) | $PM_{10}$ (μg/m³) |
|---|---|---|---|---|---|---|---|---|---|---|
| **Number of cases** | - | 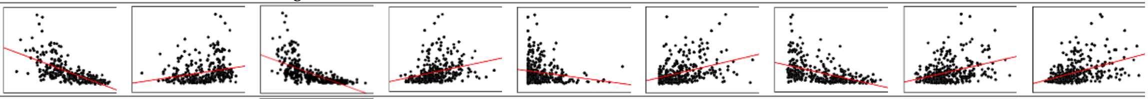 | | | | | | | | |
| **Temperature (°C)** | -0.78 *** | - | 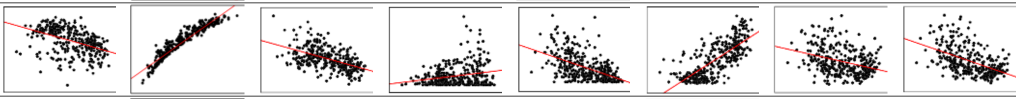 | | | | | | | |
| **Relative humidity (%)** | 0.33 *** | -0.5 *** | - | 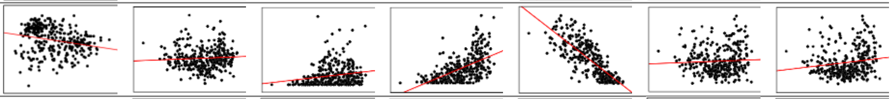 | | | | | | |
| **Absolute humidity (g/m³)** | -0.77 | 0.97 | -0.29 | - | 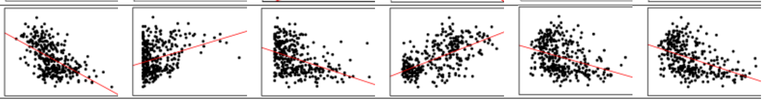 | | | | | |
| **Wind speed (m/s)** | 0.43 *** | -0.52 *** | 0.13 * | -0.54 *** | - | 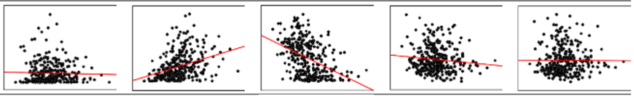 | | | | |
| **Precipitation (mm)** | -0.15 ** | 0.15 ** | 0.23 *** | 0.25 *** | *NS* | - | 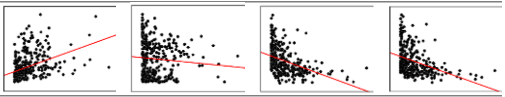 | | | |
| **Precipitation duration (hours)** | 0.42 *** | -0.55 *** | 0.61 *** | -0.43 *** | 0.39 *** | 0.58 *** | - | 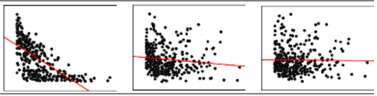 | | |
| **Sunshine duration (hours)** | -0.57 *** | 0.75 *** | -0.82 *** | 0.62 *** | -0.43 *** | -0.17 ** | -0.73 *** | - | 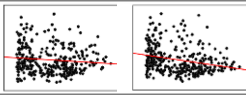 | |
| **$PM_{2.5}$ (μg/m³)** | 0.4 *** | -0.34 *** | *NS* | -0.37 *** | *NS* | -0.51 *** | -0.2 *** | *NS* | - | 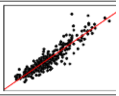 |
| **$PM_{10}$ (μg/m³)** | 0.51 *** | -0.54 *** | 0.22 *** | -0.55 *** | *NS* | -0.46 *** | *NS* | -0.29 *** | 0.92 *** | - |

Abbreviations: $PM_{2.5}$, particulate matter with aerodynamic diameters of 2.5 μm or less; $PM_{10}$, particulate matter with aerodynamic diameters of 10 μm or less

* indicates $p < 0.05$; ** indicates $p < 0.01$; *** indicates $p < 0.001$;

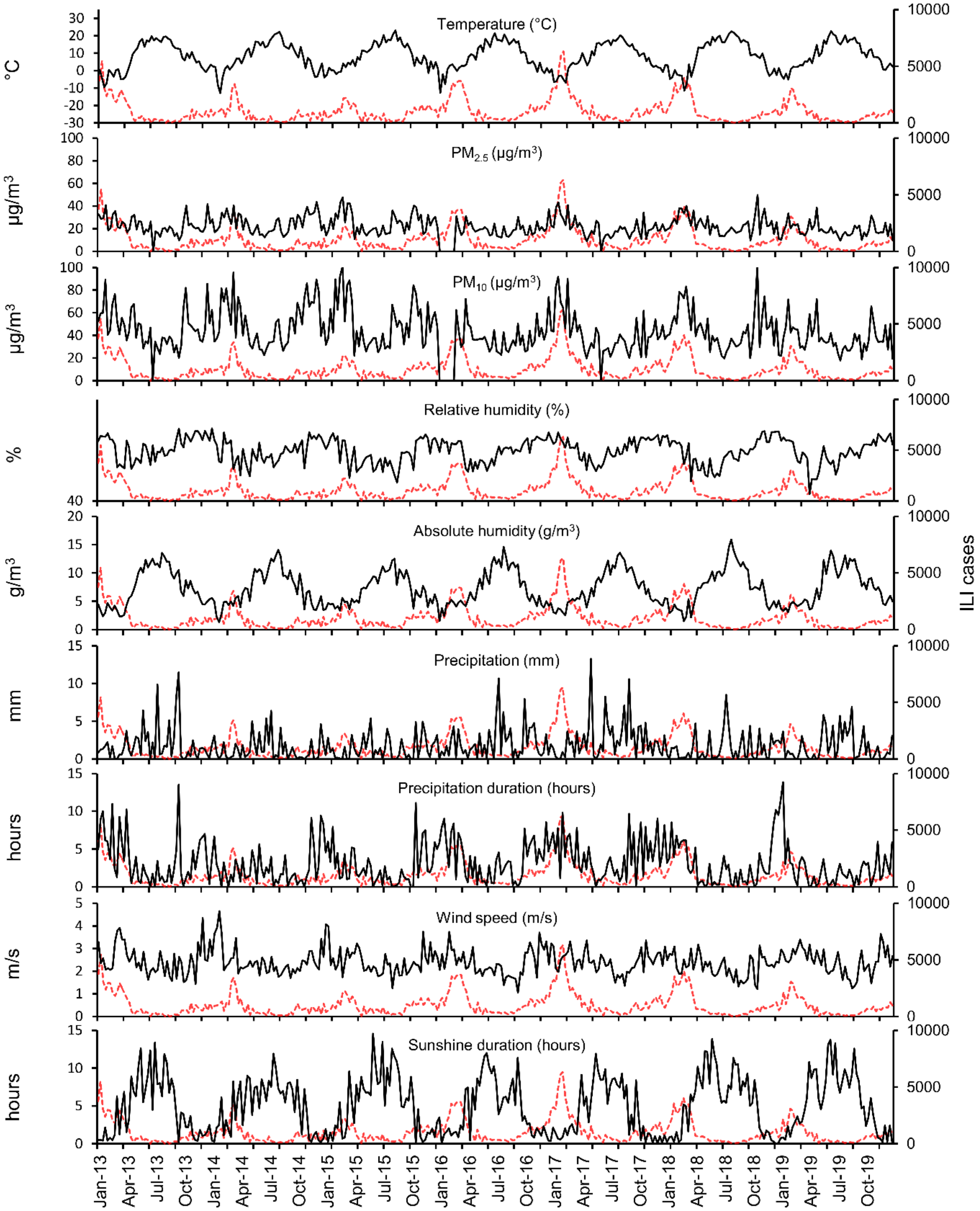


Figure S1. The number of weekly influenza-like cases (red dotted lines) plotted against meteorological and air pollution data (black lines) from Bialystok, Poland, from January 2013 – December 2019. Abbreviations: $PM_{2.5}$, particulate matter with aerodynamic diameters of 2.5 µm or less; $PM_{10}$, particulate matter with aerodynamic diameters of 10 µm or less; ILI, influenza-like illness

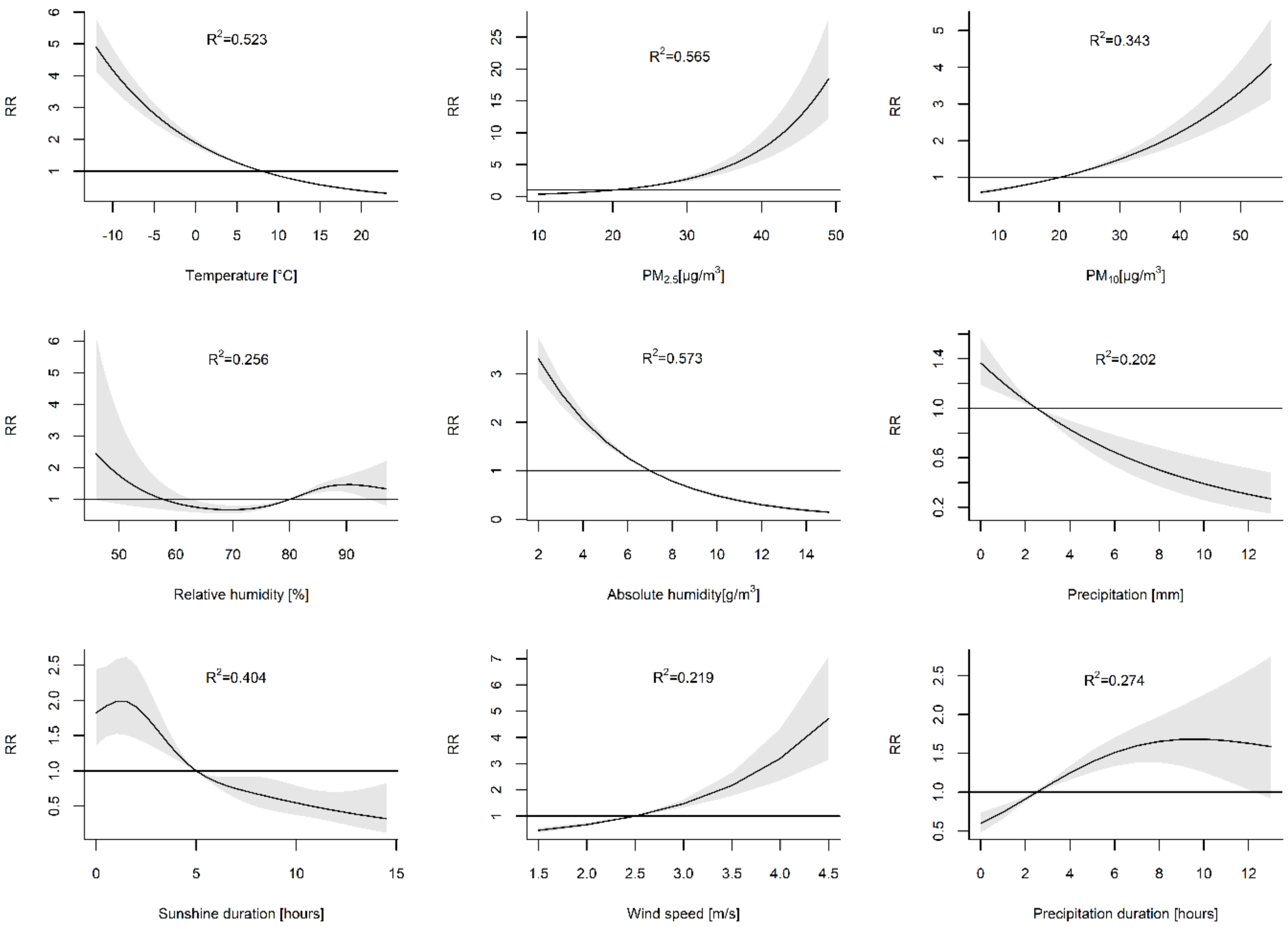


Figure S2. The association between environmental factors and the relative risk (RR) with 95% confidence interval (CI) of influenza-like illness. The analysis was performed for each environmental factor separately. The reference levels were defined as the median values of each of the analyzed variables to calculate RRs. Abbreviations: $PM_{2.5}$, particulate matter with aerodynamic diameters of 2.5 μm or less; $PM_{10}$, particulate matter with aerodynamic diameters of 10 μm or less

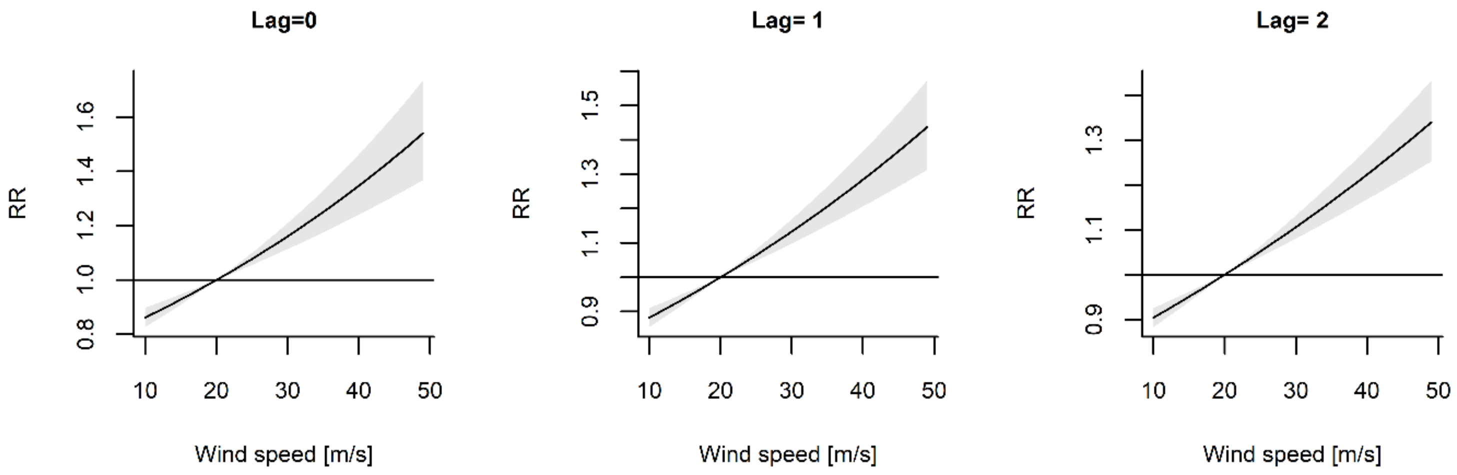


Figure S3. The association between wind speed and the relative risk (RR) of the incidence of influenza-like illness is shown as the estimated exposure-lag-response curves for lags of 0 to 2 weeks with 95% confidence intervals. The model was adjusted for the $PM_{2.5}$ concentration and a long-term trend.
