# Supplementary figures and images for: Cumulative Effects of Particulate Matter Pollution and Meteorological Variables on the Risk of Influenza-Like Illness
Source: Viruses. 2021 Mar 26;13(4):556. doi: 10.3390/v13040556 (PMC8065612; doi:10.3390/v13040556)

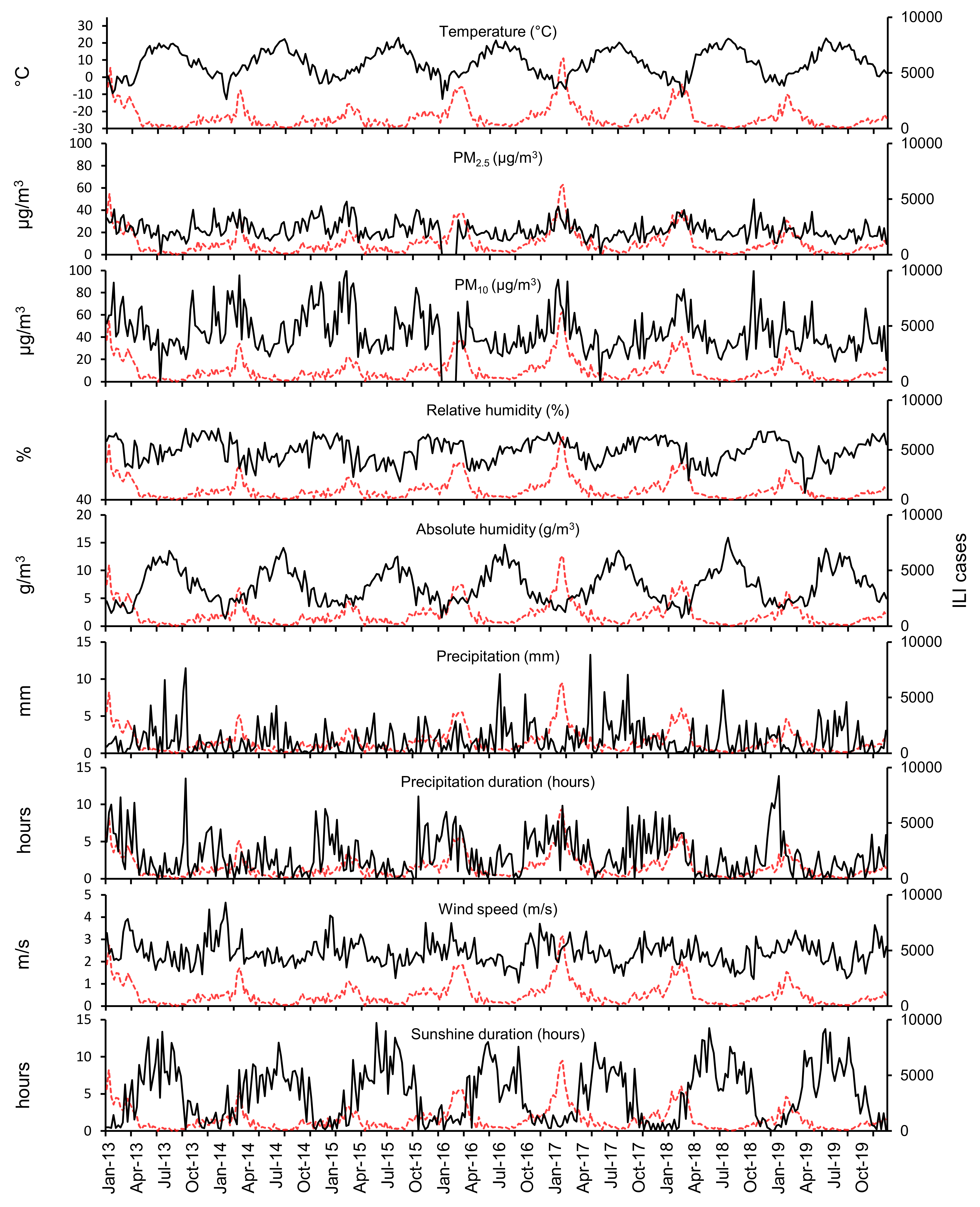

Supplement: Supplementary file 1 [file viruses-13-00556-s001.zip › Figure S1_HiRes.png]

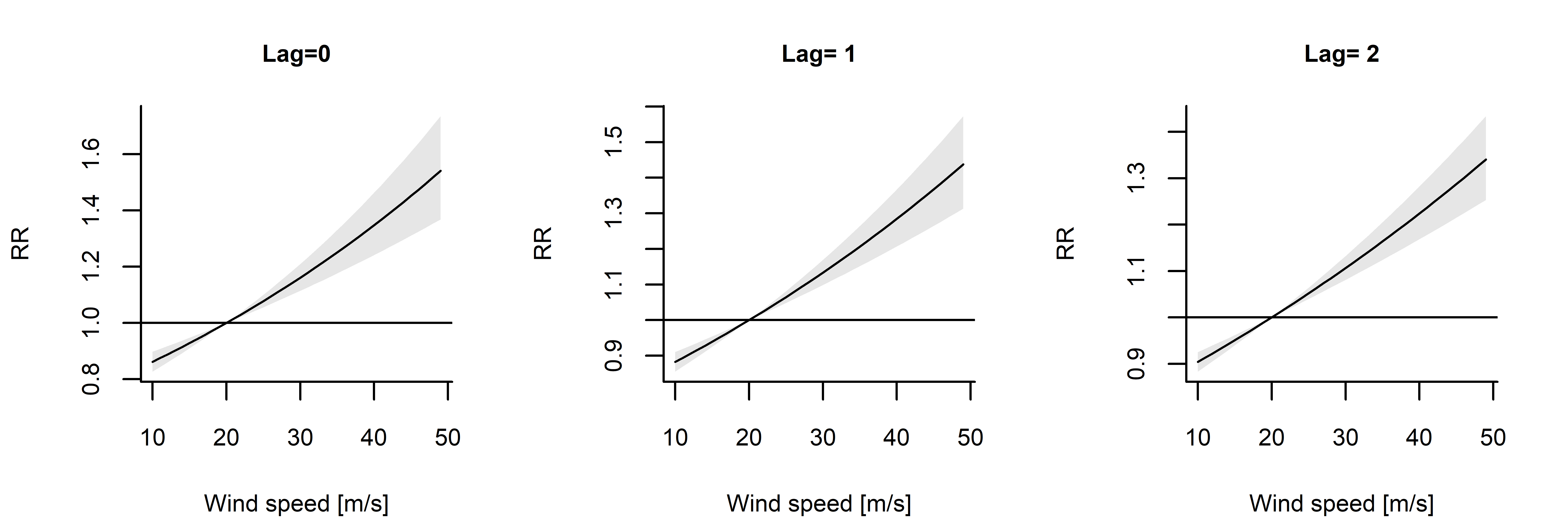

Supplement: Supplementary file 1 [file viruses-13-00556-s001.zip › Figure S3_HiRes.tif]
